# Potential molecular mechanism underlying cardiac fibrosis in diabetes mellitus: a narrative review

**DOI:** 10.1186/s43044-023-00376-z

**Published:** 2023-06-12

**Authors:** Muhammad Ridwan, Herlina Dimiati, Maimun Syukri, Ronny Lesmana

**Affiliations:** 1 Doctorate School of Medical Science, Faculty of Medicine, Universitas Syiah Kuala, Banda Aceh, 23116 Indonesia; 2 Department of Pediatrics, Faculty of Medicine, Universitas Syiah Kuala, Banda Aceh, 23111 Indonesia; 3 Department of Internal Medicine, Faculty of Medicine, Universitas Syiah Kuala, Banda Aceh, 23111 Indonesia; 4 Physiology Division, Department of Biomedical Science, Faculty of Medicine, Universitas Padjadjaran, Sumedang, West Java 45363 Indonesia

**Keywords:** Cardiac fibrosis, Heart failure, Diabetic cardiomyopathy, microRNA, TGF-β1

## Abstract

**Background:**

Diabetes mellitus (DM) is among the most common risk factors for cardiovascular disease in the world with prevalence of more than 500 million population in 2021. Cardiac fibrosis with its complex process has been hypothesized as one of the mechanisms explaining development of heart failure in diabetic patients. Recently, the biomolecular mechanism of cardiac fibrosis in the hyperglycemia setting has been focusing around transforming growth factor β-1 (TGFβ-1) as a major factor. However, there is interplay role of several factors including microRNAs (miRNAs) which acts as a potential regulator of cardiac fibrosis connected with TGFβ-1. In this review, we explored interplay role of several factors including microRNAs which acts as a potential regulator of cardiac fibrosis connected with TGFβ-1 in diabetes mellitus. This narrative review included articles from the PubMed and Science Direct databases published in the last 10 years (2012–2022).

**Main text:**

In diabetic patients, excessive activation of myofibroblasts occurs and triggers pro-collagen to convert into mature collagen to fill the cardiac interstitial space resulting in a pathological process of extracellular matrix remodeling. The balance between matrix metalloproteinase (MMP) and its inhibitor (tissue inhibitor of metalloproteinase, TIMP) is crucial in degradation of the extracellular matrix. Diabetes-related cardiac fibrosis is modulated by increasing level of TGF-β1 mediated by cellular components, including cardiomyocyte and non-cardiomyocyte cells involving fibroblasts, vascular pericytes smooth muscle cells, endothelial cells, mast cells, macrophages, and dendritic cells. Several miRNAs such as miR-21, miR-9, miR-29, miR-30d, miR-144, miR-34a, miR-150, miR-320, and miR-378 are upregulated in diabetic cardiomyopathy. TGF-β1, together with inflammatory cytokines, oxidative stress, combined sma and the mothers against decapentaplegic (smad) protein, mitogen-activated protein kinase (MAPK), and microRNAs, is interconnectedly involved in extracellular matrix production and fibrotic response. In this review, we explored interplay role of several factors including microRNAs which acts as a potential regulator of cardiac fibrosis connected with TGFβ-1 in diabetes mellitus.

**Conclusions:**

Long-term hyperglycemia activates cardiac fibroblast via complex processes involving TGF-β1, miRNA, inflammatory chemokines, oxidative stress, smad, or MAPK pathways. There is increasing evidence of miRNA’s roles lately in modulating cardiac fibrosis.

## Background

Diabetes mellitus has become a worldwide health concern due to the rise of sufferers to 537 million in 2021 [1]. According to the World Health Organization (WHO), diabetes was recorded as the ninth leading cause of death, with about 1.5 million deaths in 2019 [2]. The uncontrolled hyperglycemic conditions can lead to fatal cardiovascular complications, such as hypertension [3, 4], stroke [5], atherosclerosis [6, 7], myocardial infarction [8, 9], atrial fibrillation [10], cardiac fibrosis [11–14], diabetic cardiomyopathy [15–17], and sudden cardiac death [18–20]. Moreover, the significant increase in diabetes mellitus prevalence raises awareness of cardiovascular system complications [1], in which heart rhythm disturbances [21] and heart failure [22, 23] are the most common cardiovascular complications resulting from prolonged high blood sugar levels.

Diabetes-induced heart failure is a result of the pathological stimulus of prolonged hyperglycemia leading to cardiac remodeling [24]. In general, cardiac remodeling involves changes of the myocardium structure, metabolism, and electrical conduction of the heart as a result of stimulation of endogenous and exogenous factors, which in turn will cause structural changes and cause biological effects on the heart ventricles [25]. Typical features of diabetes-induced cardiac remodeling are endothelial changes, cardiomyocyte hypertrophy and apoptosis, and extracellular matrix accumulation leading to cardiac fibrosis. Initially, the accumulation of extracellular matrix is actually a beneficial protective mechanism for wound healing and tissue regeneration. However, when the extracellular matrix collagen accumulation occurs excessively and continuously, over time, it will cause disturbances in tissue function [24]. The hypothetical mechanism that is thought to underlie the accumulation of extracellular matrix in patients with diabetes mellitus is a decrease in the activity of an enzyme responsible for the degradation of the extracellular matrix, namely matrix metalloproteinase (MMP) and the resultant increase in tissue inhibitor of metalloproteinase (TIMP) [26, 27]. In the case of diabetes, type III collagen is expressed more than type I collagen [28].

In addition to hyperglycemic conditions, the presence of advanced glycation end products (AGEs), reactive oxygen species (ROS), and neurohumoral activation triggers the activation of cardiac fibroblast cells, induces a proliferative response, and triggers the synthesis of matrix phenotypes. Secretion of fibrogenic growth factors and modulation of fibroblast phenotype can also be stimulated by cardiomyocytes and endothelial cells. High plasma concentrations of inflammatory cytokines due to release by adipose cell hypertrophy in diabetes result in systemic inflammation and activation of endothelial cells that will stimulate monocyte infiltration and transformation into macrophages. Activated macrophages will produce transforming growth factor-β (TGF-β) which plays an important role in fibroblast stimulation and initiates the secretion of pro-fibrotic mediators [5].

TGF- β is a prototype factor in the form of a strong profibrotic cytokine and has an active role in the formation of fibrosis, including cardiac fibrosis [29, 30]. In other words, TGF-β is one of the general mediators that play a role in the development of fibrosis formation. For example, in cases of cardiac injury, modulation of profibrotic mediators such as TGF-β, angiotensin II, endothelin-1 will activate cardiac fibroblasts resulting in proliferation and migration of cardiac fibroblasts. This situation will also trigger extracellular matrix protein deposition and myofibroblast differentiation to form cardiac fibrosis. It is therefore believed that inhibiting TGF-β signaling, either by molecular inhibition, antibody, or genetic deletion, will provide a favorable outcome for the treatment of cardiac fibrosis [31].

In addition to the case of heart injury, other cases that trigger the formation of cardiac fibrosis will also increase TGF-β. Studies with animal models have shown that both type-1 diabetes mellitus (T1DM) and type-2 diabetes mellitus (T2DM) still experience an increase in TGF-β [32, 33]. On the other hand, inhibiting TGF-β will reduce the incidence of cardiac fibrosis [14].

Interestingly, cardiac fibrosis has recently been associated with cytokine secretion by cardiac fibroblasts and cardiomyocytes modulated by microRNAs (miRNAs) [34]. miRNA is a small (22–26 nucleotide) single-stranded endogenous non-coding RNA whose function is to control the expression of target messenger RNAs (mRNAs). The expression can be either mRNA degradation or translational repression [35]. The biological role is formed from direct binding to the target 3'UTR mRNA so that it can affect the stability and translation of mRNA [36] by regulating mRNA expression in the transcriptional phase [37]. In its role, miRNA affects the fibrosis process positively and negatively depending on the gene to which it binds. Some miRNAs trigger antifibrotic activity by inhibiting the signaling activity of the TGF-β component in myofibroblasts, but on the other hand, the remaining miRNAs play a pro-fibrotic role by upregulating the expression of TGF-β signaling molecules, one of which is miR-21 [38]. Unfortunately, there is unclear cut explanation about role of diabetes mellitus-induced heart failure involving TGF-β and microRNA stimulation. Taken together, this review proposes a wider comprehensive aspect of molecular mechanism explaining interrelated hypothetical pathways of TGFβ-1, hyperglycemia, and miRNA.

This narrative review included articles from the PubMed and Science Direct databases published in the last 10 years (2012–2022). The search used a combination of medical subject heading (MeSH) terms, i.e., [(cardiac fibrosis) OR (cardiac remodeling) OR (myocardial fibrosis)] AND [(diabetes mellitus) OR (type-2 dm) OR (type-1 dm) OR (hyperglycemia)]. We excluded review articles and book chapters; then, articles met eligibility criteria were further evaluated meticulously by screening each article.

## Main text

### The advantages and disadvantages of fibrosis for the heart

Cardiac fibrosis is a condition where scar tissue is formed due to the accumulation of collagen deposition, as well as activation and differentiation of cardiac fibroblasts into myofibroblasts [39]. When an injury appears due to cardiac disorders, such as myocardial infarction, hypertensive heart disease, hypertrophic diabetic cardiomyopathy, or idiopathic dilated cardiomyopathy, the heart exhibits a self-repairing response by remodeling or regeneration using the remaining cells [39–41]. An extracellular matrix deposition is a protective mechanism for wound repair and tissue regeneration. The reason is that collagen compensates for cells that die due to injury; therefore, at a particular level, collagen's role is highly beneficial because it maintains cardiac structural integrity and protects from cardiac rupture. However, a massive and continuous process can be detrimental and even lead to death. Excessive collagen deposition is found to cause heart damage and to impair cardiac compliance because it leads to increased cardiomyopathy and reduced cardiac elasticity due to the formation of scar tissues [42–44]. The formation of cardiac fibrosis as a pathological process due to extracellular matrix remodeling leads to abnormalities in the composition and quality of the extracellular matrix. This condition will affect the cardiac function, i.e., decreasing the ejection fraction due to myocardial matrix stiffness from fibrotic injuries, disruption of the cardiac electrical conduction, and eventually death [41].

## Fibrosis mechanism in diabetic patients

In diabetic patients, scar tissue is formed in the cardiac interstitial cells due to long-term hyperglycemic conditions through a pathological process of extracellular matrix remodeling [13, 24, 39, 40, 45].

### Cardiac fibroblast activation and myofibroblasts

Fibroblasts are cells that have a significant role in the formation of cardiac fibrosis [46]. Under normal conditions, fibroblasts are in a resting or inactive state; however, they can be activated by the remaining healthy cardiac tissue whenever cardiac cell injury occurs [47].

After the activation, fibroblasts are called myofibroblasts, which are pro-inflammatory, hypersecretory, and hypermigratory. Myofibroblasts have anti-inflammatory and pro-angiogenic phenotypes; thus, they can express cytokines, extracellular matrix, and other paracrine factors used in wound healing due to the injuries [48].

### Collagen deposition

Dynamic regulation of the extracellular matrix plays a vital role in maintaining cardiac structural integrity. A disturbance in the regulation of extracellular matrix synthesis, collagen deposition, and extracellular matrix degradation will induce the pathogenesis of cardiac fibrosis. Precursor cells will be triggered to activate myofibroblasts in diabetic patients with long-term uncontrolled hyperglycemia. Excessive activation of myofibroblasts triggers pro-collagen to convert into mature collagen, expressing type I and type III collagen to fill the cardiac interstitial space [10]. The expressed collagen then cross-links with lysyl oxidase to produce fibers that inhibit protein degradation. Type I collagen is related to stiffness, while type III collagen is related to wall tension elasticity. Collagen turnover involves several types of cells, i.e., inflammatory and mediator cells, such as cytokine cells, growth factors, and hormones [49, 50]. These factors help in myofibroblast differentiation and extracellular matrix remodeling [50].

### Cardiac fibrosis

Cardiac fibrosis is a pathological condition occurring as an excess accumulation of extracellular matrix due to cardiac remodeling process involving profibrotic cells, growth factors, and expression of inflammatory cytokines. A higher level of profibrotic phenotype in fibroblasts was found in diabetic patients than in non-diabetic patients [10]. Myofibroblast activation is the main effector in cardiac fibrosis; however, monocytes, lymphocytes, mast cells, vascular cells, and cardiomyocytes also produce a fibrotic response by expressing fibrinogen mediators. The related mediators include inflammatory cytokines and chemokines, ROS, mast cell-derived proteases, endothelin-1, renin–angiotensin–aldosterone-system (RAAS), cellular components, and growth factors such as TGF-β [51]. The aspects that cause cardiac fibrosis affect the nature of fibrosis, e.g., the resulting cardiac fibrosis is more localized in myocardial infarction. Meanwhile, in diabetic cardiac fibrosis, the fibrosis is formed more diffusely and is located in the cardiac interstitial part. There are several types of cardiac fibrosis based on the cause. Replacement fibrosis is formed due to myocardial infarction, sarcoidosis, myocarditis, toxic cardiomyopathy, and chronic renal insufficiency. Infiltrative interstitial fibrosis is present in amyloidosis and Anderson–Fabry disease. Meanwhile, reactive interstitial fibrosis is formed in hypertension, diabetes, non-ischemic dilated cardiomyopathy, hypertrophic cardiomyopathy, sarcoidosis, and chronic renal insufficiency [43, 44].

The myocardial interstitial space is a cardiac tissue compartment consisting of stromal cells and an extracellular matrix complex composed of structural and non-structural proteins and bioactive signaling molecules. Structural proteins include collagen and elastin, while non-structural proteins include glycoproteins, proteoglycans, and glycosaminoglycans. The extracellular matrix is structurally complex, dynamic, and organized, thus, maintaining structural integrity, cardiac cycle transmission, communication signals between cells, and repair response after cardiac injury [52].

### Histopathology of cardiac fibrosis

**Fig. 1:**
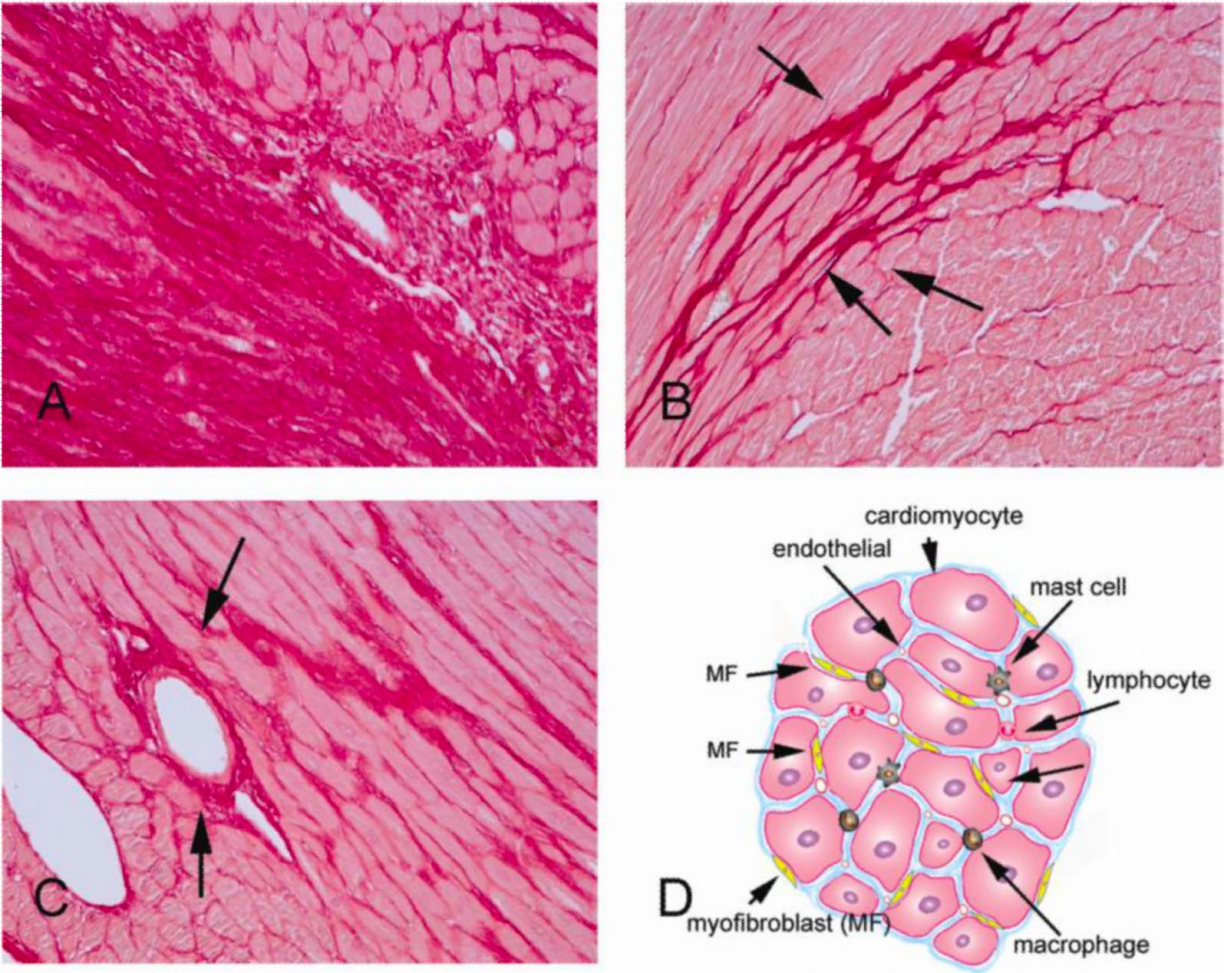
Different types of cardiac fibrosis based on histopathological features of model rat preparations using Sirius-Red staining. **A** Replacement fibrosis is characterized by the sudden loss of large numbers of cardiomyocytes, mostly caused by myocardial infarction. **B** Interstitial fibrosis is characterized by increased collagen deposition in the cardiac interstitial space due to significantly reduced cardiomyocyte cells. **C** Perivascular fibrosis is characterized by expansion of the vascular adventitia matrix. **D** Illustration of cardiac fibrosis in the cardiac interstitial space influenced by the primary effector cells, i.e., myofibroblasts, and other mediators that play a role, such as macrophages, lymphocytes, mast cells, vascular endothelial cells, and cardiomyocytes. With written permission from Frangogiannis NG [51]

The formation of perivascular and interstitial fibrosis in diabetic patients has been studied through histopathological studies, and its process is independent of the pathogenesis of coronary atherosclerosis and hypertension [53]. Histological findings of the left ventricular endomyocardial biopsy revealed increased cardiac fibrosis formation and deposition of advanced glycation end products in heart failure with reduced ejection fraction (HFrEF). Meanwhile, an increase in cardiomyocyte resting tension was discovered in heart failure with preserved ejection fraction (HFpEF) [54] (Fig. 1).

### Cardiac remodeling

Diabetes-related cardiac remodeling can cause various manifestations referring to the restrictive or dilated left ventricle. Compared to non-diabetic conditions, there is an increase in cardiac hypertrophy, narrow left ventricular end-diastolic and end-systolic volume indices, higher E/E’ ratio, and an increased prevalence of left ventricular geometry abnormalities, such as concentric left ventricle remodeling, concentric hypertrophy, and eccentric hypertrophy in diabetes [55].

The change in cardiac structure due to diabetic condition shows different features in HFrEF and HFpEF. In HFrEF, there is an increase in cardiac cell death followed by fibrotic scar tissue formation triggered by the stimulation of protein kinase C in cardiac fibroblasts in hyperglycemic situations [56]. The remodeling process in diabetic cardiac fibrosis involves the interaction of profibrotic cells, growth factors, and the expression of inflammatory cytokines [53]. Lipotoxicity occurs in diabetic conditions due to triglyceride or fatty acid accumulation [56]. In addition, deposition of advanced glycation end products that trigger inflammation, immune cell infiltration, and subsequent apoptosis also happens in diabetes condition. Meanwhile, in HFpEF, cardiac cell stiffness and hypertrophy occur due to hyperinsulinemia and endothelial dysfunction, a microvascular complication of diabetes mellitus [57].

## Factors influencing cardiac fibrosis

### Cellular components

Diabetes-related cardiac fibrosis is mediated by cellular components, including cardiomyocyte and non-cardiomyocyte cells that bind to each other in the interstitial matrix involving fibroblasts, vascular pericytes smooth muscle cells, endothelial cells, mast cells, macrophages, and dendritic cells [58].

Fibroblasts are the primary cells that play a role in forming diabetic-associated cardiac fibrosis [59]. After myocardial infarction or myocardial remodeling, fibroblasts differentiate into myofibroblasts, express contractile proteins such as α-smooth muscle actin (α-SMA), and synthesize extracellular proteins [60].

Monocytes and macrophages are abundant in the myocardium when cardiac fibrosis develops, triggering a fibrogenic phenotype to activate fibroblasts. In type I and type II diabetes, monocytes and macrophages have contributed to fibrotic remodeling through fibrogenic mediation [61]. Lymphocyte cells could also modulate the fibroblast phenotype and trigger fibrosis during myocardial remodeling [62].

Endothelial and pericytes cells are known to contribute to the formation of cardiac fibrosis. Endothelial-to-mesenchymal transition (EndMT) helps increase fibroblast activation. In diabetic conditions, EndMT expands in the cardiac interstitial space. Pericytes cells modulate cardiac fibrosis by increasing myofibroblast conversion and through vascular cells secrete mediators that activate fibroblasts [63]. Mast cells are thought to produce proteases and fibrogenic growth factors in the formation of cardiac fibrosis. In diabetic conditions, delaying mast cell accumulation affects healing [64, 65].

Cardiomyocytes, in diabetic conditions, release toxic substances that are damaging and can cause cell death. Fibrosis in diabetic conditions is in the form of scar tissue that replaces damaged or dead cardiomyocytes due to the released toxic substances. In addition, in hyperglycemic conditions, fibrogenic phenotypes in cardiomyocytes are expressed, such as triggering the synthesis and release of growth factors and cytokines, which stimulate the proliferation and activation of fibroblasts. Cardiomyocytes also produce pro-inflammatory mediators that help activate immune cells and trigger the formation of fibrotic tissue [58].

### Mitogen-activated protein kinases

Mitogen-activated protein kinases (MAPKs) play a significant role in biological processes, i.e., proliferation, differentiation, metabolism, motility, cell survival, and cell apoptosis. MAPKs consist of four subfamilies, namely extracellular-signal-regulated kinase 1/2 (ERK 1/2), Jun N-terminal Kinase (JNK), p38, and extracellular-signal-regulated kinase 5 (ERK 5). Currently, MAPKs are one of the targets of pharmacological therapy or genetic manipulation of heart development, heart function, and heart disease [66].

Studies with genetic manipulation of JNK1 deletions have led to increased formation of cardiac fibrosis [67]. Similarly, studies using JNK1 inhibitor therapy in hamster cardiomyopathy models show increased apoptosis and cardiac fibrosis [68]. Another in-vivo study shows that increased JNK activity through loss of β1-integrins is associated with increased MMP-2 activity rather than MMP-9, which lead to reduced cardiac fibrosis formation [69].

The p-38 MAPK also demonstrated a similar thing. During tissue stress and cardiac remodeling, myocardial p-38 activation results in restrictive cardiomyopathy followed by a significant increase in the amount of interstitial fibrosis [70]. MAPK p-38 is also thought to stimulate the release of pro-inflammatory cytokines in myocytes, such as tumor necrosis factor-α (TNF-α), interleukin-6 (IL-6), TGF-β, and vascular cell adhesion molecules-1 (VCAM-1) which affects cardiac remodeling through regulation of proliferation, differentiation, and function of immune cells [71, 72].

### Smad protein

Activation of smad-2 and smad-3 as R-smad is primarily significant in fibrotic infiltration of fibroblasts, while smad-1,5,8 is not yet understood. Smad-3 affects the formation of cardiac fibrosis by stimulating myofibroblast phenotype activation, extracellular matrix synthesis, integrin expression, and secretion of proteases and anti-proteases [73]. Smad-3 also stimulates myofibroblast conversion, increases the expression of α-SMA protein, and stimulates the transcription of extracellular matrix structure, including regulating type I collagen, type III collagen, fibronectin, periostin, and tenascin-C, in vitro. In addition, smad-3 reduces MMP expression and stimulates TIMP expression [73–75]. Meanwhile, Smad-2 helps modulate fibronectin and periostin. Smad-2 and Smad-3 together affect fibroblast gene transcription, as well as nuclear activation and translocation. Meanwhile, smad 6, as an i-smad, inhibits the response of bone morphogenetic protein (BMP), while smad-7 inhibits both TGF-β and BMP [73].

Smad is a group of proteins that serves as intracellular mediators against the binding formed by TGF-β on the cell surface. Smad activation initiates translocation from the cytoplasm to the nucleus. Along with transcription factors, it plays an important role in transcriptional regulation to regulate target gene expression. Smad combines sma protein and the mothers against decapentaplegic (mad) gene [76, 77]. Based on its function, smad is classified into three groups, i.e., receptors activated smad (R-smads), common smad (Co-smad), and inhibitory smad (i-smad). R-smad, responsible for stimulating TGF-β signaling, consists of smad-1, smad-2, smad-3, smad-5, and smad-8. Co-smad, which binds to R-smad and helps form a signaling complex, consists only of smad-4. Meanwhile, i-smad, a negative R-smad regulator, consists of smad-6 and smad-7 [78].

### Transforming growth factor-β 1

TGF-β, expressed from activated macrophages, is a cytokine that plays a role in forming fibrosis in various tissues, including cardiac tissue [79]. TGF-β plays a role in the activation of myofibroblast infiltration and is responsible for extracellular matrix production and fibrotic response. This process increases left ventricular diastolic stiffness leading to HFpEF [5].

TGF-β1 is the dominant isoform in the cardiovascular system, which is more abundant than other TGF-β isoforms. In cases of cardiac fibrosis, it is only limited to TGF-β1. In a healthy heart condition, TGF-β1 is expressed in a latent form and is not associated with its receptors; however, when a heart injury occurs, conversion of TGF-β1 occurs from the latent to the active form. Only a small amount of activated TGF-β1 is required to induce the cellular response system. Various mediators have been appointed as TGF-β1 activators in cardiac fibrosis, one of which is MMP-2. In contrast with MMP, TIMP inhibits MMP performance [51].

In vitro administration of empagliflozin, a sodium glucose co-transporter 2 inhibitor (SGLT2i) as an oral antidiabetic drug, was able to reduce TGF-β expression, fibroblast activation and extracellular matrix remodeling, and the formation of profibrotic markers, such as collagen type I alpha I (COL1A1), actin alpha 2 (ACTA2), αSMA, connective tissue growth factor (CTGF), fibronectin-1 (FN1), and MMP2 [80]. Empagliflozin exerts an antifibrotic effect by inhibiting the TGFβ-Smad pathway. A low dose of canagliflozin, another variant of SGLT2i, also exerted antifibrotic effects by reducing fibronectin, collagen, and TGF-1 in db/db mice [81, 82]. TGF-β is predicted to modulate miR-21 by targeting Jagged 1 and Smad7. TGF-β accelerates the division of pri-miR-21 into pre-miR-21, thereby increasing mature miR-21 [83].

### Matrix metalloproteinase

Matrix metalloproteinase (MMP) regulates the degradation of the extracellular matrix. The human body expresses 23 types of MMPs classified according to their constituent substrates. The collagenase group includes MMP-1, MMP-13, and MMP-8; the gelatinase group includes MMP-2 and MMP-9; stromelysins or matrilysins groups include MMP-3 and MMP-7; the membrane group includes MMP-14 [84]. MMP-2 is a specific marker for the fibroblast population [85].

In diabetic patients, the MMP2 activity, a regulator of enzyme activity that plays a role in extra-cellular-matrix (ECM) degradation, changes [52]. In its application, MMP-2 is used as a diagnostic model for left ventricular hypertrophy development and cases of developing heart failure (HF) [86, 87]. A study using an animal model discovered that genetic deletion of MMP-2 resulted in a reduction in the accumulation rate of myocardial collagen fibrils and improved the left ventricular diastolic function [88]. MMP-2 expression is thought to affect ECM stability, induce ECM structural changes, and improve profibrotic signaling pathways [84].

It has been reported that diabetic-associated hypertrophy is blocked by inhibiting the expression of TGF-β1 receptor signaling or inhibiting MMP2 and MMP9 in vitro. Preventing activation of MMP2 or MMP9 inhibits cell hypertrophy induced by high sugar levels [89].

### Tissue inhibitor of metalloproteinase

In contrast with MMP, TIMP is an MMP inhibitor that plays a significant role in heart fibrosis and HF processes [90]. An imbalance between MMP and TIMP leads to cardiac fibrosis and heart failure [49]. There are four groups of TIMP with high affinity for MMP binding, namely TIMP-1, TIMP-2, TIMP-3, and TIMP-4. Initially, it was thought that the TIMPs had similar functional characteristics; however, each of the TIMPs had specific functions [84]. TIMP-1 is predicted to influence the collagen degradation process associated with interstitial myocardial fibrosis [52]. An increased TIMP-1 affects MMP expression and reduces ECM degradation [91, 92].

### Advanced glycation end products and its receptors

Advanced glycation end products (AGEs) are accumulated under prolonged exposure to hyperglycemia. This condition induces collagen cross-linking that can increase the formation of cardiac fibrosis, followed by increased cardiac rigidity and impaired cardiac relaxation [93]. AGEs increase the production of ROS and receptors for advanced glycation end products (RAGE) [94]. A prolonged hyperglycemic condition triggers the neutrophil derivative of calcium-binding protein S100 A8/A9 (S100A8/A9) in its interaction with Kupffer cells and triggers the release of IL-6. SGLT2-i helps inhibit the bond formed between S100A8/A9 and RAGE [95]. A low dose of SGLT2-i (Empagliflozin 10 mg/kg/day) did not significantly affect the reduction in AGE and ROS or the formation of RAGE. However, a threefold dose of empagliflozin showed reduced expression of AGE, RAGE, and ROS [94].

### Inflammation

Long-term hyperglycemia increases plasma concentrations of inflammatory cytokines and activates intracellular cardiomyocyte cytokines. The systemic inflammation and activation of these endothelial cells stimulate monocyte infiltration to transform into macrophages, which also play a role in ECM overproduction [5].

Chemokines are chemotactic cytokines that affect leukocytes and are involved in the fibrotic process. The chemokine C–C ligand 2 (CCL2) or monocyte chemotactic protein-1 (MCP-1) is primarily associated with heart disease, including ischemic heart and cardiac fibrosis. It was discovered that MCP-1 could reduce cardiac fibrosis and improve diastolic dysfunction without affecting blood pressure or systolic function but did not reduce cardiomyocyte hypertrophy [96].

### Oxidative stress

Diabetic patients tend to experience long-term exposure to oxidative stress, which plays a role in various complications, including cardiovascular-related [97]. In long-term hyperglycemia, mitochondria of the cardiac cells are unable to cope with the conditions, increasing the ROS and apoptotic cascade [98].

ROS directly regulates the quantity and quality of the interstitial extracellular matrix through modulation of matrix protein expression and metabolism. Increased oxidative stress can activate MMP and decrease fibrillar collagen synthesis in cardiac fibroblasts. ROS also increases extracellular matrix deposition in the cardiac interstitial via TGF-β activation. As a mediator, ROS plays a role in the induction of cytokines and the effect of angiotensin II on fibroblasts. ROS is also involved in the activation of transcription factors, such as activator protein-1 (AP-1), E26 transformation specific (ETS) transcription factor, and nuclear factor kappa-light chain enhancer of activated B cells (NF-kB), which helps MMP transcription [51]. The induction of oxidative stress in cardiac tissue is triggered due to increased cardiomyocyte intracellular inflammatory cytokines. Increased oxidative stress also increases the expression of inducible nitric oxide synthase (iNOS), triggers cell death, and increases the accumulation of ECM proteins, thereby increasing protein rigidity in cardiac muscle cells [5].

### Renin–angiotensin aldosterone system

Exposure to hyperglycemia also increases the RAAS activation, causing overproduction of angiotensin II and aldosterone, which triggers cardiac hypertrophy, cardiac fibrosis, and impaired cardiac relaxation. In adult mice, it was discovered that angiotensin II modulates collagen production by fibroblasts via ROS generation. In addition, mitochondrial oxidative stress is understood to mediate fibroticity through angiotensin induction [51, 93, 99].

### Micro-ribonucleic acid (micro-RNA, miRNA)

Expression of miRNA almost constantly changes in every step of the biological process. miRNAs are small single-stranded non-coding RNAs that regulate gene expression at the post-transcriptional level [100]. miRNAs are broadly expressed in the nucleus and cytoplasm. However, miRNAs can still be found in blood circulation [101]. As miRNAs are expressed stably in blood, they are likely to be used as potential biomarkers for diagnostic, prognostic, and novel therapeutic strategies related to cardiovascular disease [102]. It has been reported that in diabetic conditions, miRNAs expression is dysregulated in serum/plasma [100]. A number of miRNAs were recorded in plasma and whole blood in diabetic conditions; those are miR-21, miR-320, miR-15a, miR24, miR29b, miR-20b miR-126, miR150, miR197, miR-223, miR191, miR486, miR-28-3p [93], miR-9, miR-29a, miR-30d, miR34a, miR124a, miR-146a, miR375, miR-144, and miR192 [103, 104].

**Fig. 2:**
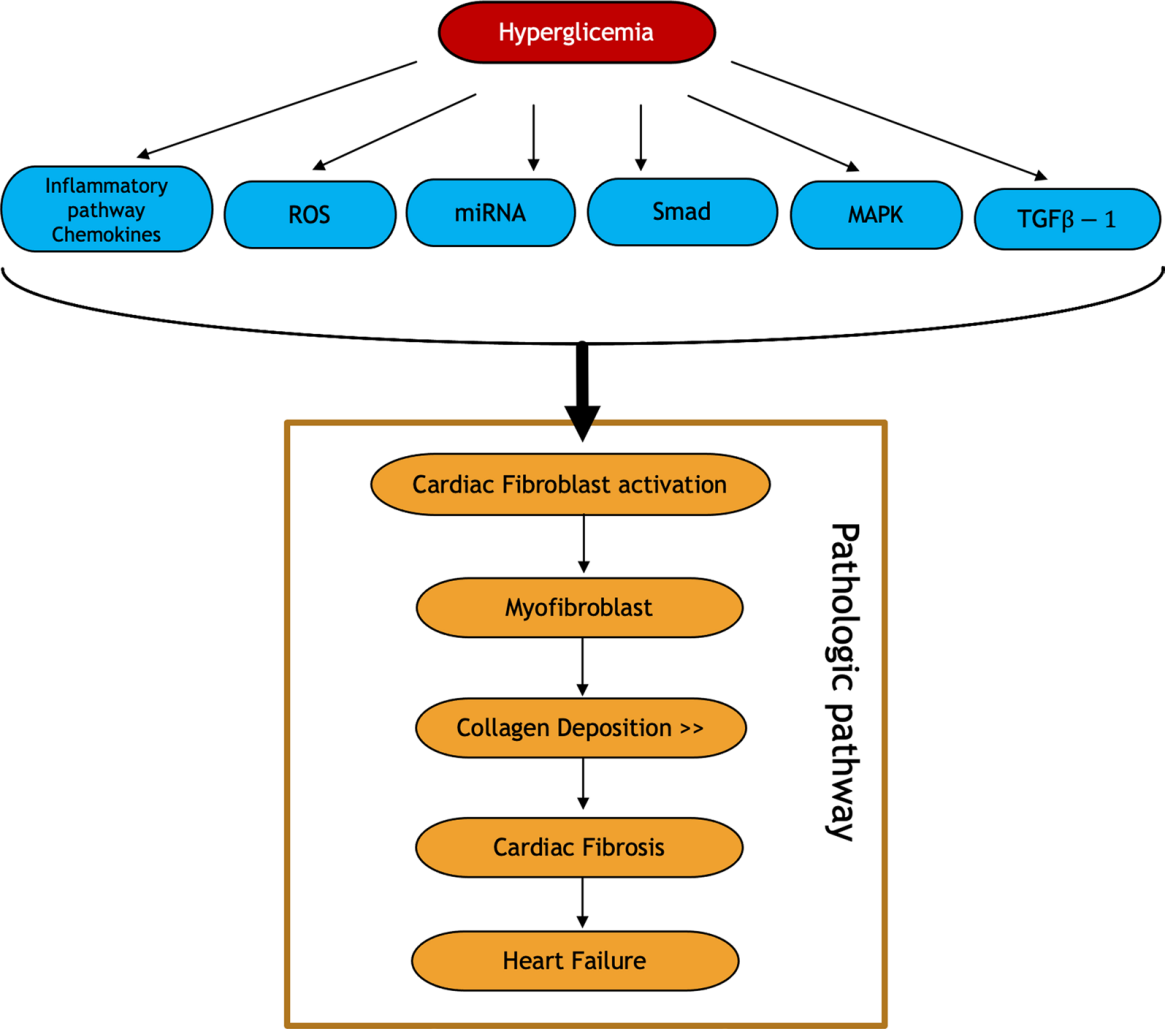
Interrelated biological mechanism schematic pathways of cardiac fibrosis in hyperglycemia setting lead to heart failure. Long-term hyperglycemia affects cardiac fibroblast activation via inflammatory chemokines, ROS, miRNA, Smad, MAPK, or TGF-β pathways. Cardiac fibrosis is resulted from the increased collagen deposition in the extracellular matrix leading to heart failure

Nine recorded miRNAs were upregulated related to diabetic cardiomyopathy, namely miR-21, miR-9, miR-29, miR-30d, miR-144, miR-34a, miR-150, miR-320, and miR-378. Interestingly, miR-21 was acted via a pro-fibrosis pathophysiological mechanism [102]. miR-21 suppresses the expression of pro-viral integration sites for Moloney murine leukemia virus-1 (Pim1) and B-cell lymphoma-2 (Bcl-2), which are anti-apoptotic and cardioprotective proteins. It increases profibrotic markers, such as TGF-β, collagen, and fibronectin[82]. Overexpression of MiR-21 in heart failure modulates the activation of extracellular signal-regulated kinase mitogen-activated protein kinase (ERK-MAPK) by inhibiting sprouty protein homolog 1 (SPRY1). In addition, the increase in miR-21 leads to changes in cardiofibroblast (CF) viability and increases the accumulation of factors that induce hypertrophy, thereby modulating cardiac remodeling. Increased miR-21 modulates the activation of fibrotic remodeling by inhibiting protein phosphatase and tensin homolog (PTEN) in cardiac fibrosis [83] (Fig. 2).

## Implication in future cardiac fibrosis therapy

To date, various therapeutic regimens have been used in cardiac fibrosis [41, 105]. For over decades, angiotensin-converting enzyme inhibitors (ACEi) [106], angiotensin receptor blocker (ARB) [107], and mineralocorticoid receptor antagonist (MRA) [108] have been used as heart failure treatment by targeting the RAAS as anti-remodeling drugs [106–108]. As a combination, the angiotensin receptor blocker neprilysin inhibitor (ARNI) is considered to reduce interstitial fibrosis and cardiomyocyte hypertrophy in diabetes [109, 110].

The use of SGLT2-i is one of the many promising regimens for cardiac fibrosis despite its original function as an oral antidiabetic drug. As of 2021, heart failure guideline by European Society of cardiology (ESC), followed by American Heart Association guideline in 2022, has included SGLT2-i as one of 4 compulsory drug classes for HFrEF patients to reduce hospitalization and cardiovascular mortality independence of type 2 diabetes presence [111, 112].

However, further study of the biomolecular mechanisms in SGLT2-i is necessary, considering that the SGLT2 receptor is not expressed in the heart [41, 105]. Besides, another promising therapeutic target for cardiac fibrosis has been studied in mice by adding oligonucleotide to bind with certain miRNA (anti-miRNA) [49, 113]. Therefore, new directions for targeted therapy in cardiac fibrosis are needed.

## Conclusions

The currently reported biological mechanism of diabetes-associated cardiac fibrosis remains unclear, i.e., reported through the fragments of overlapping pathways. This review provides a hypothesis of the overall pathway and the association between each pathway. The pathway includes the critical roles of TGFβ-1, microRNA, MAPK, ROS, smad protein, and related inflammatory pathways. The hypothesis of the complete pathway is expected to generate further studies, such as potential effective therapies for cardiac fibrosis patients.

## Data Availability

Data sharing is not applicable to this article as no datasets were generated or analyzed during this literature review.

## Abbreviations

ACEi: Angiotensin-converting enzyme inhibitors
ARB: Angiotensin receptor blocker
ARNI: Angiotensin receptor blocker neprilysin inhibitor
ACTA2: Actin alpha 2
AGEs: Advanced glycation end products
BMP: Bone morphogenetic protein
CCL-2: Chemokine C–C ligand-2
COL1A1: Collagen type i alpha I
CTGF: Connective tissue growth factor
DM: Diabetes mellitus
ECM: Extracellular matrix
EndMT: Endothelial-to-mesenchymal transition
ERK: Extracellular signal-regulated kinase
ERK/MAPK: Extracellular signal-regulated kinase/mitogen-activated protein kinase
FN1: Fibronectin-1
HF: Heart failure
HFrEF: Heart failure with reduced ejection fraction
HFpEF: Heart failure with preserved ejection fraction
iNOS: Inducible nitric oxide synthase
JNK: C-jun n-terminal kinase
MAPK: Mitogen-activated protein kinase
MCP-1: Monocyte chemotactic protein-1
mRNAs: Messenger RNA
miRNA: Micro-RNA
miR-21: Micro-RNA-21
MMP: Matrix metalloproteinase
MRA: Mineralocorticoid receptor antagonist
PTEN: Protein phosphatase and tensin homolog
RAAS: Renin angiotensin aldosterone system
RAGE: Receptor advanced glycation end products
ROS: Reactive oxygen species
SCD: Sudden cardiac death
SGLT: Sodium glucose co-transporter
SGLT2-i: Sodium glucose co-transporter 2 inhibitor
α-SMA: α-Smooth muscle actin
Spry1: Sprouty homolog-1
TIMP-1: Tissue inhibitor of metalloproteinase type 1
TNF-α: Tumor necrosis factor-α
TGF-β1: Transforming growth factor-β1
TIMP-1: Tissue inhibitor of metalloprotein-1
VCAM-1: Vascular cell adhesion molecules-1
WHO: World Health Organization
